# Computational approaches for detecting protein complexes from protein interaction networks: a survey

**DOI:** 10.1186/1471-2164-11-S1-S3

**Published:** 2010-02-10

**Authors:** Xiaoli Li, Min Wu, Chee-Keong Kwoh, See-Kiong Ng

**Affiliations:** 1 Institute for Infocomm Research, 1 Fusionopolis Way, Singapore; 2 School of Computer Engineering, Nanyang Technological University, Singapore

## Abstract

**Background:**

Most proteins form macromolecular complexes to perform their biological functions. However, experimentally determined protein complex data, especially of those involving more than two protein partners, are relatively limited in the current state-of-the-art high-throughput experimental techniques. Nevertheless, many techniques (such as yeast-two-hybrid) have enabled systematic screening of pairwise protein-protein interactions *en masse*. Thus computational approaches for detecting protein complexes from protein interaction data are useful complements to the limited experimental methods. They can be used together with the experimental methods for mapping the interactions of proteins to understand how different proteins are organized into higher-level substructures to perform various cellular functions.

**Results:**

Given the abundance of pairwise protein interaction data from high-throughput genome-wide experimental screenings, a protein interaction network can be constructed from protein interaction data by considering individual proteins as the nodes, and the existence of a physical interaction between a pair of proteins as a link. This binary protein interaction graph can then be used for detecting protein complexes using graph clustering techniques. In this paper, we review and evaluate the state-of-the-art techniques for computational detection of protein complexes, and discuss some promising research directions in this field.

**Conclusions:**

Experimental results with yeast protein interaction data show that the interaction subgraphs discovered by various computational methods matched well with actual protein complexes. In addition, the computational approaches have also improved in performance over the years. Further improvements could be achieved if the quality of the underlying protein interaction data can be considered adequately to minimize the undesirable effects from the irrelevant and noisy sources, and the various biological evidences can be better incorporated into the detection process to maximize the exploitation of the increasing wealth of biological knowledge available.

## Introduction

Most proteins form complexes to accomplish their biological functions [1-3]. In fact, it is well known that many proteins exist as parts of permanent obligate complexes. For example, the all-important hemoglobin molecule is actually a permanent assembly of four globular protein subunits [4]. Many enzymes are also multisubunit assemblies that fold and bind simultaneously. Even transient interactions such as hormone-effector and signaling-effector interactions are also involved the formation of protein complexes [5]. Biologically, protein complexes are the key molecular entities to perform many essential biological functions, such as the transcription of DNA, the translation of mRNA, signal transduction, cell cycle and so on. For example, the RNA polymerase II complex transcribes genetic information into messages for ribosomes to produce proteins [6]; the Proteasome core particle is a large barrel-like complex containing "core" of four stacked rings around a central pore. It is involved in the degradation of proteins, which is an essential process within the cell [7]; the nuclear pore complex is responsible for the protected exchange of components between the nucleus and cytoplasm and for preventing the transport of material not destined to cross the nuclear membrane [8].

While there are a number of ways to detect protein complexes experimentally, Tandem Affnity Purification (TAP) with mass spectrometry [9] is the preferred experimental detection method used by many research groups. However, there are several limitations to this method [10]. For example, its multiple washing and purification steps tend to eliminate transient low affnity protein complexes. Also, the tag proteins used in the experiments may interfere the protein complex formation. Gavin *et al. *[2] have shown that TAP-MS only captures limited known yeast protein complex subunits. Furthermore, in TAP-MS the subcellular location of complexes is lost due to the *in vitro *purification of whole-cell lysates [11]. This means that time-consuming preparation of subcellular fractionated lysates may be needed for a less-studied cellular process in order to employ subcellular localization information to validate the experimental results and detect false negatives or false positives. Due to these experimental limitations, alternative computational approaches for detecting the complexes are thus useful complements to the experimental methods for detecting protein complexes.

Recently, high-throughput methods (e.g. yeast-two-hybrid [12,13]) for detecting pairwise protein-protein interactions (PPIs) *en masse *have enabled the construction of PPI networks on a genomic scale. A graphical map of an entire organism's interactome can be constructed from such experiments by considering individual proteins as the nodes, and the existence of a physical interaction between a pair of proteins as a link between two corresponding nodes. Given that protein complexes are molecular groups of proteins that work together as "protein machines" for common biological functions, we may expect the protein complexes to be functionally and structurally cohesive substructures in the binary PPI networks [14]. Researchers have recently begun to explore this concept to help discover new protein complexes. The main line of these researches is based on the observation that densely connected regions in the PPI networks often correspond to actual protein complexes [15], suggesting the identities of protein complexes can be revealed as tight-knitted substructures in protein-protein interaction maps [16]. However, as PPI networks are large-scale graphical data consisting of tens of thousands of pairwise protein-protein interactions, sophisticated graph clustering techniques have to be proposed to handle the computational challenge. In this paper, we will review the state-of-the-art techniques to mine protein complexes from protein interaction networks. We will describe classical graph clustering for complex mining as well as some new emerging techniques. We will also present the evaluation metrics that are commonly used by researchers in evaluating their approaches. Using these evaluation metrics, we will perform a comparative study of the various methods to evaluate the state-of-the-art techniques. In addition, we will discuss some promising future research directions in the field. Of course, the ultimate success of the protein complex detection from PPI networks will depend on the parallel improvements both in the experimental techniques by biologists to provide rich and reliable biological data sets for computational data mining, and in the graph mining techniques by computer scientists to provide effcient and robust ways to fully exploit the protein interaction data to discover new knowledge.

## Methods

Before we review the current computational approaches for protein complex detection, let us make a principled distinction between two biological concepts, namely, protein complexes and functional modules [17]. A protein complex is a physical aggregation of several proteins (and possibly other molecules) via molecular interaction (binding) with each other at the same location and time. A functional module also consists of a number of proteins (and other molecules) that interact with each other to control or perform a particular cellular function. However, unlike protein complexes, these proteins do not necessarily interact at the same time and location. In this review, we do not distinguish protein complexes from functional modules because the underlying protein interaction data that we are using for protein complex detection do not provide temporal and spatial information. To distinguish between protein complexes and functional modules, it will be necessary to integrate additional biological resources that contain such information (e.g., gene expression data) [18] .

Let us now introduce some terminologies which are widely used in protein complex mining. Then, we will present the use of traditional graph clustering techniques for complex mining followed by some new emerging techniques for this task.

### Terminologies

A PPI network is often modeled as an undirected graph *G *= (*V,E *), where *V *is the set of nodes (proteins) and *E *= {(*u, v *)|*u, v *∈*V *} is the set of edges (protein interactions). A graph *G*_1_ = (*V*_1_*,E*_1_) is a subgraph of *G *if and only if *V*_1_⊆*V *and *E*_1_⊆*E*. For a node *v *⊆*V *, the set of *v *'s direct neighbors is denoted as *N_v_* where *N_v_* = {*u *|*u *⊆*V*, (*u, v *) ⊆ *E *}. *v *'s degree in *G*, *deg *(*v *), is the cardinality of *N_v_*, i.e., |*N_v_*|. *Density. *

The density of the graph *G*, denoted as *density *(*G *), is defined to quantify the richness of edges within *G *as shown in equation (1) [19]. Basically, 0 ≤ *density *(*G *) ≤ 1. If *density *(*G *) = 1, then *G *is the fully connected graph or a clique, which has the maximum number of edges, i.e., every pair of distinct vertices is connected by an edge.(1)

*Clustering Coeffcient. *The clustering coeffcient of a node *v *is the density of the subgraph formed by *N_v_* and their corresponding edges, which quantifies how close *v *'s neighbors are to being a clique (complete graph) [20].

*Local Neighborhood. *Given a node *u *∈*V*, its local neighborhood graph *G_u_* is the subgraph formed by *u *and all its immediate neighbors with the corresponding interactions in *G *. It can be formally defined as 

*G_u_*= (*V_u_, E_u_*), where *V_u_*= {*u *} ∪* N_u_*, and *E_u_*= {(*v_j_ , v_k_* )|(*v_j_ , v_k_* ) ∈* E, v_j_ , v_k_* ∈* V_u_*}

#### Weighted PPI networks

If the edges in the PPI network are weighted (e.g., weights represent the reliability of protein interactions [21,22]), the definitions of vertex degree, density can be extended to their corresponding weighted versions as follows in equation (2):

(2) and ,					

where *w *(*e *) is the weight of the edge *e *.

Similarly, the weighted clustering coeffcient of the node *v *is the weighted density of the subgraph formed by *Nv *and their corresponding edges.

### Graph clustering for protein complex mining

First, we review the conventional graph clustering approaches for protein complex mining. These methods mine for cliques or densely connected subgraphs in PPI networks which could correspond to protein complexes. While the methods mainly use the PPI networks for mining, additional information, such as gene expression data [23,24], functional information (e.g., Gene Ontology data [25]) as well as other biological information [26], may also be exploited to enhance the quality of predicted complexes.

#### Graph clustering based solely on PPI networks

In this section, let us describe the graph clustering approaches that use PPI networks as the sole underlying dataset for the mining task.

**Figure 1:**
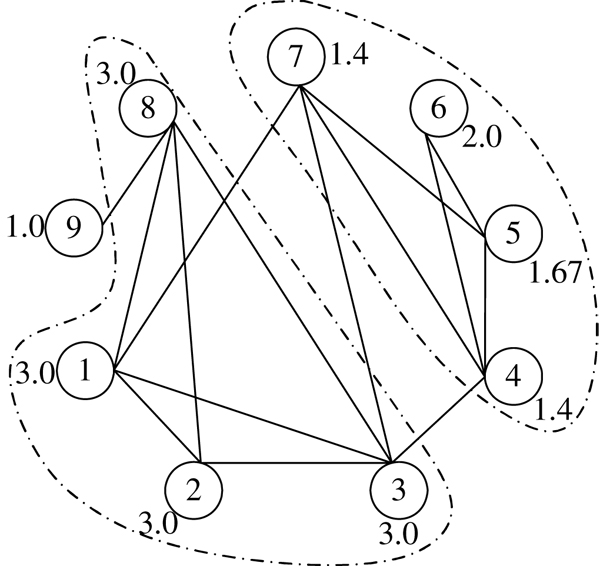
An example of how MCODE detects protein complexes from a small sample graph of protein-protein interactions.

***MCODE.***The MCODE algorithm proposed by Bader *et al. *[27] is one of the first computational methods to detect protein complexes based on the proteins' connectivity values in the PPI network. MCODE first weighs every node based on their local neighborhood densities, and then selects seed nodes with high weights as initial clusters and augments these clusters by outward traversing from the seeds. In addition, MCODE has an optional post-processing step with operations such as filtering non-dense subgraphs and generating overlapping clusters. Figure 1 shows an example of how MCODE detects protein complexes from a small sample graph of protein-protein interactions. The sample PPI graph consists of 9 nodes (proteins), which are labeled from 1 to 9. MCODE first assigns each node a weight using its local neighborhood density. For example, the node set {4, 5, 6, 7} is the highest *k*-core in node 5's neighborhood graph with *k*=2 [28,29] and density *d *= 5/6 (5 interactions out of a total possible 6 interactions between the nodes). Thus, node 5 has an initial weight *w *(5) = *k *× *d *= 2 × 5/6 = 1.67. Next, the node with the highest weight (without loss of generality, node 1 is first selected here) is selected as an initial cluster. Node 2, as node 1's neighbor, satisfies the weight constraint to be included into the cluster because *w *(2) = 3 ≥ (1 – *T_w_*) × *w *(1). Here *T_w_* is a threshold for cluster formation that is set as 0.2 by default. Similarly, nodes 3 and 8 are also added into this cluster and finally MCODE predicts {1, 2, 3, 8} as a protein complex. Subsequently, {4, 5, 6, 7} is detected as another putative protein complex from this sample PPI graph.

The experimental results of MCODE method showed that the number of predicted complexes is generally small and the size of many predicted complexes is often too large.

***Clique.*** Spirin and Mirny [17] proposed three methods for protein complex prediction from PPI network. The first method is to exhaustively enumerate the full cliques (fully connected subgraphs) as protein complexes. However, the use of cliques was too constraining given that the incompleteness in the PPI data. As such, they also applied the Super-Paramagnetic Clustering (SPC) and a Monte Carlo (MC) simulation for the same purpose. Their experiments show MC performed better than SPC for clusters that share common nodes and for high density graphs, whereas SPC has an advantage identifying clusters that have very few connections to the rest of the graph. The MC algorithm has a drawback in that the size of the predicted clusters needs to be pre-defined by users.

***MCL.*** Markov Clustering (MCL) [30,31] can also be applied to detect functional modules and protein complexes by simulating random walks in PPI networks. MCL manipulates the weighted or unweighted adjacency matrix with two operators called expansion and inflation. The expansion operator assigns new probabilities for all pairs of nodes, while the inflation operator changes the probabilities for all these walks in the graph, boosting the probabilities of intra-cluster walks and demoting inter-cluster walks. Iterative expansion and inflation will separate the PPI network into many segments as protein complexes. Due to its robustness [32], MCL is also applied to detect protein complexes from the pull-down data [33-36].

***LCMA.*** Instead of adopting the over-constraining full cliques as the basis for protein complexes, Li *et al. *[37] devised an LCMA algorithm (Local Clique Merging Algorithm) that adopts a local clique merging method as an attempt to address the current incompleteness limitation of protein interaction data. For each protein, LCMA effciently locates a local clique in its neighborhood graph in the first step. In the second step, LCMA merges local cliques that share high similarity (with large overlaps) as protein complexes. Evaluation results show that LCMA was more effcient and effective in detecting complexes than the full clique method [17] described above.

***DPClus.*** Amin *et al. *[38] proposed a cluster periphery-tracking algorithm (DPClus) to detect protein complexes by keeping track of the periphery of a detected cluster. DPClus first weighs each edge based on the common neighbors between its two proteins and further weighs nodes by their weighted degree. To form a protein complex, DPClus first selects the node with the highest weight (seed node) as the initial cluster and then iteratively augments this cluster by including vertices one by one, which are out of but closely related with the current cluster.

***PCP.*** Chua *et al. *[39] proposed an algorithm, ProteinComplexPrediction (PCP), for complex prediction. PCP first applies FS-weights [40] to evaluate the reliability of protein interactions and then modifies the PPI network by removing interactions with low FS-weights and including novel indirect interactions with high FS-weights. In the modified PPI network, PCP detects and merges dense subgraphs as protein complexes, using an effcient clique-finding algorithm from [41] and a partial clique merging.

***Hub Duplication.*** Ucar *et al. *[42] developed a refinement method to detect protein complexes in scale-free PPI networks. Hub proteins (with degree greater than 25 in [42]) are first selected and their neighborhood graphs are subsequently constructed. A hub-duplication strategy is then applied to detect dense subgraphs in these neighborhood graphs with multi-functional hub proteins assigned to multiple clusters.

***CFinder.*** Adamcsek *et al. *[43] provided a software called CFinder to find functional modules in PPI networks. CFinder detects the *k*-clique percolation clusters as functional modules using a *Clique Percolation Method *[44]. In particular, a *k*-clique is a clique with *k *nodes and two *k*-cliques are adjacent if they share (*k *– 1) common nodes. A *k*-clique percolation cluster is then constructed by linking all the adjacent *k*-cliques as a bigger subgraph.

***SCAN.*** Mete *et al. *[45] proposed a new methodology called SCAN to detect functional modules in PPI networks. SCAN is extended from a well-known density-based clustering called DBSCAN [46]. SCAN first defines the structural similarity between two proteins based on their common neighbors. Two proteins are structure-reachable if their structural similarity is greater than a threshold and a protein is a core node if it has several structure-reachable neighbors. SCAN augments a cluster from a core node by iteratively including structure-reachable neighbors. Additionally, SCAN can also identify the hubs and outliers in the PPI networks.

***GS.*** Navlakha *et al. *[47] applied a graph summarization (GS) technique [48] to cluster a PPI graph into functional modules. GS compresses the input PPI graph into a summary graph which shows a high-level structure of the input graph. In the summary graph, the nodes correspond to non-overlapping sets of proteins which share similar interacting neighbors in the PPI network and thus are predicted as functional modules.

***CMC.*** Liu *et al. *[22] recently proposed a Clustering method based on Maximal Cliques (CMC) to detect protein complexes. CMC first obtains all the maximal cliques by applying a maximal clique mining algorithm [41]. CMC then assigns each interaction a score based on the reliability measure in [49]. Therefore, each clique can be scored with its weighted density. Last, CMC removes or merges highly overlapping cliques to generate protein complexes. In particular, if two cliques are highly overlapping, CMC either merges these two cliques as a bigger one or simply removes the one with a lower score (weighted density) depending on their inter-connectivity.

### Incorporating gene expression data

Proteins which interact with each other can be expected to be activated and repressed under the same conditions. In other words, interacting proteins are likely to exhibit similar gene-expression profiles. In fact, gene expression data has been widely exploited to annotate protein functions (guilt by association) and predict novel protein-protein interactions [50-52]. We describe below some methods for incorporating gene-expression data to help identify protein complexes in PPI networks.

***GFA.*** Feng *et al. *[53] proposed a graph fragmentation algorithm (GFA) to detect protein complexes using protein interaction graphs weighted with microarray data. For a PPI graph *G *= (V, E), two different density definitions as shown in equation (3) are used:

(3) and ,					

where *w *(*v *) is the weight of the protein *v *weighted by *e^–expression^*^ (*v *)^, and *expression *(*v *) is the log fold change of *v*'s gene-expression profile. GFA first applies DSA (Densest Subgraph Algorithm) [54] to find the densest subgraphs by maximizing the ratios in equation (3). It then removes redundant subgraphs occurring in different samples of the microarray data. The resulting clusters are genes that are highly differentially co-expressed and hence likely to be protein complexes.

***DMSP.*** Maraziotis *et al. *[55] developed an algorithm called “Detect Module from Seed Protein” (DMSP). DMSP operates in three phases. First, proteins are clustered based on the gene-expression data using fuzzy c-means algorithm. Given two proteins from different clusters, their similarity is calculated by the distance between two cluster centroids and the distance from each protein to its corresponding centroid. Second, the extensions of weighted degree and density are obtained for seed selection and dense subgraph formation. Third, given a seed protein *s*, its neighbors and even its indirect neighbors are iteratively included based on different criteria to form the module.

***MATISSE.*** Ulitsky *et al. *[56] also proposed a method called MATISSE (Module Analysis via Topology of Interactions and Similarity SEts) to grow a functional module from a set of seed proteins. First, the edge weights are mainly determined by the gene expression correlation between interacting proteins and the node weights are their weighted degree. Second, given a protein with the highest weight, *k *neighbors with the highest weights are picked to form a set of (*k *+ 1) seeds. Last, after selecting the seeds, Jointly Active Connected Subnetworks (JACS) are obtained by several operations (e.g., adding proteins into the sets of seeds). Two small JACSs are merged to form a new one if they are closely connected.

### Incorporating functional information

Functional information can also be incorporated to accurately detect protein complexes. Since proteins within the same protein complex are generally aggregated to perform a common function, the functional enrichment of a cluster can be used to indicate its tendency to be a real complex. The reliability of interactions, evaluated by the consistency of functional similarity between two proteins, can also help to provide cleaner PPI data for protein complex detection.

***RNSC.*** King *et al. *[57] proposed a “Restricted Neighborhoods Search Clustering” (RNSC) algorithm to detect protein complexes based on both graph-theoretical and gene-ontological properties. RNSC starts with an initial random clustering and then searches for a better clustering with the minimum costs by vertex-moving. RNSC discards unpromising clusters based on their size, density and functional homogeneity. The functional homogeneity of a cluster is defined as the smallest p-value over all the functional groups. Relatively few complexes are predicted by RNSC and its results depended heavily on the quality of the initial clustering which is random or user-defined.

***DECAFF.*** Li *et al. *[16] proposed an algorithm called DECAFF (Dense-neighborhood Extraction using Connectivity and conFidence Features) to incorporate functional information to detect dense and reliable subgraphs as protein complexes. Firstly, a Hub-removal algorithm is developed to mine multiple possibly overlapping dense subgraphs in a neighborhood graph. Secondly, the dense subgraphs detected by the Hub-removal algorithm and the local cliques by LCMA [37] are processed by a merging operation if two subgraphs have a large overlap. Thirdly, DECAFF filters away possible false protein complexes with low reliability, ensuring that the proteins in the predicted protein complexes are connected by high confidence protein interactions in the underlying network. Here, the reliability of a subgraph is the average reliability of the edges within a complex, which is estimated by using a probabilistic model with the functional information of interacting proteins.

***SWEMODE.*** Lubovac *et al. *[58] presented an algorithm called SWEMODE (Semantic WEights for MODule Elucidation) to detect functional modules in PPI networks. SWEMODE assigns the weights to the nodes in a different manner. First, each edge is weighted by the semantic similarity of GO terms annotating its two proteins [59-62]. Second, the nodes have two different weighting schemes based on their weighted clustering coeffcients. Finally, SWEMODE selects seeds and augments the clusters from the seeds in a similar way as MCODE [27].

***STM.*** Cho *et al. *[63] extended flow-based modularization approach called STM [64] to identify functional modules and protein complexes by considering the functional information. In this work, two novel measures are developed to index the reliability of interactions based on GO terms. The weights of proteins are their corresponding weighted degree. Informative proteins (those with large weights) are then selected and the flow simulation from each informative protein will decompose the weighted PPI network into preliminary clusters. A post-processing step is also devised to merge similar preliminary clusters as protein complexes.

### Using TAP datasets for mining

The techniques discussed above have used pairwise physical interactions detected by high-throughput experiments such as Y2H as the PPI dataset for detecting protein complexes. More recently, there are some researchers who attempt to detect protein complexes from interaction data obtained solely from TAP experiments. Unlike Y2H method which detects direct physical interactions, using TAP data requires careful weighing of the detected links as TAP also detects indirect interactions in protein complexes. Krogan *et al. *[33] were one of the first to use high-throughput purification data to predict protein complexes. In their constructed PPI network, the edge weights are learned by machine learning techniques from both the purification records and the mass spectrometry scores. Markov Clustering (MCL) [30,31] is then applied to generate non-overlapping clusters as protein complexes.

Caroline *et al. *[36] proposed a novel method to detect protein complexes by bootstrap sampling. First, several bootstrap samples (1000 in this work) are selected from the purification records with replacement. Then, MCL [30,31] is applied to generate preliminary complexes from each sample with edge weights using socio-affinity indices [3]. Finally, protein complexes are detected by MCL in the bootstrap network, where two proteins have an edge if they are clustered together in at least one sample. The reliability of an edge in the bootstrap network is inferred by the number of samples for which two proteins are in the same preliminary complex.

Pu *et al. *[34] also applied MCL to detect protein complexes from the purification data. In their work, the reliability of interactions are inferred from a scoring function, which combines the evidence in each purification for bait-prey and prey-prey relationships [65]. Similarly, Hart *et al. *[35] used alternative scoring scheme together with MCL to detect protein complexes.

**Figure 2:**
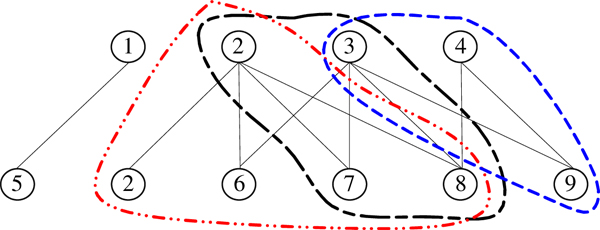
CODEC to detect protein complexes from TAP data which are modeled as a bipartite graph.

Recently, Geva *et al. *[66] proposed a new approach called CODEC to detect protein complexes from TAP data. Unlike above methods that convert TAP data to PPI networks, CODEC models the TAP data as a bipartite graph *G *= (*U, V, E *) where *U *and *V *represent the sets of baits and preys respectively and *E *describes the bait-prey relationships detected in the experiments as shown in Figure 2. CODEC defines a likelihood ratio score for a candidate bipartite subgraph to measure its density versus the chance that it is randomly generated. CODEC first identifies candidate complexes from the neighborhood of each prey protein and then modifies them by adding or deleting vertices to maximize their likelihood ratio scores. Subsequently, CODEC filters the redundant candidates and obtains the final list of protein complexes. In Figure 2, {1, 2, 3, 4} is the set of baits and {5, 2, 6, 7, 8, 9} is the set of preys in this sample bipartite graph. CODEC finally predicts three protein complexes from this graph, namely {2, 6, 7, 8}, {2, 3, 7, 8} and {3, 4, 9} with likelihood ratio scores 2.39, 2.50 and 1.79, respectively.

## Recent emerging techniques

In this section, we review a number of emerging techniques for protein complex detection that are different from the application of traditional graph clustering described in the previous section.

### Complex detection with supervised graph clustering

The previous graph clustering methods described above are unsupervised and are more or less based on the basic assumption that dense subgraphs in PPI networks are likely to be protein complexes. The protein complexes detected by many of these methods must be either cliques or defective cliques [17,67] or are dense subgraphs that have density values above a pre-selected threshold (e.g., 0.7) [16,37,38,57]. The flow-based clustering methods [30,63] are also biased towards dense subgraphs. It is possible that some protein complexes may not have a dense structure in the underlying PPI network and they will thus not be detected by these methods.

Qi *et al. *[68] proposed a supervised graph clustering framework to predict protein complexes without such prior assumptions on the topological properties of protein complexes. By using a supervised approach, important topological and biological properties of known protein complexes will be learned as guideline to detect new complexes in the PPI networks.

To obtain the training data, they collect available known protein complexes and also generate some random subgraphs as non-complexes. Topological and biological properties of these training graphs are then summarized as features. A probabilistic Bayesian network (BN) is then applied to integrate all these features and the parameters of this BN model are learned from the training data.

Given a graph candidate and its corresponding features, a log likelihood ratio score can thus be calculated by the BN model to show whether it is qualified to form a complex. A simulated annealing search [69] is further employed to modify the candidate graph if possible. For example, a new protein can be included into a candidate protein complex if the resulting augmented cluster has a higher log likelihood ratio score. Experimental results showed that the extracted features are capable to distinguish complex versus non-complexes and the supervised manner can provide more accurate identification of protein complexes. However, the knowledge learned from the limited training data could be biased and affect the complex formation during the clustering.

### Complex detection from TAP data without constructing the PPI networks

In the previous section, we have mentioned that some researchers have explored the use of TAP data instead of Y2H data for complex detection. However, as TAP does not detect direct pairwise protein-protein interactions (unlike Y2H), the PPI networks constructed using TAP data are not ideal for detecting protein complexes. Recently, several techniques are proposed to directly detect protein complexes from the TAP data without constructing the PPI networks.

Rungsarityotin *et al. *[70] applied Markov Random Fields (MRF) to detect protein complexes directly from the high-throughput TAP data. A potential function is first defined by incorporating the observation errors (the false negatives and false positives) in the purifications. False negative and false positive rates are then estimated using maximum likelihood. Finally, a Mean Field Annealing algorithm is applied to minimize the potential function and obtain the cluster assignment which is based on the MRF.

Chu *et al. *[71] used a Bayesian approach to detect protein complexes from the high-throughput affnity purification data. Protein complex memberships are represented as a matrix *Z*, where each entry *z_ij_* indicates that the *i ^th^* protein is in the complex *j *. The prior distribution of *Z *is learned from an infinite latent feature model. By considering the pairwise similarity between proteins obtained by a graph diffusion kernel [72], the posterior distribution of *Z *is further inferred to indicate the protein complex memberships by the Gibbs sampling.

### Complex detection by considering exclusive or cooperative interactions

Two adjacent interactions (those with a common protein) may be mutually exclusive [73,74] due to the overlapping binding interfaces on the common protein. A dense cluster detected by the traditional methods by blindly treating all protein-protein interactions in the PPI network to be able to occur simultaneously may be a false prediction as it may contain several mutually-exclusive interactions.

Jung *et al. *[75] recently proposed a method to extract Simultaneous Protein Interaction Clusters (SPIC) by considering this issue. They used both MCODE [27] and LCMA [37] to generate initial clusters. They then collect the SPICs by excluding the conflicting interactions so that it is possible for all the interactions within a SPIC to occur at the same time. Unfortunately, there are still many practical problems that need to be addressed, such as collecting more conflicting interactions based on 3D structure data and developing more effcient methods for SPIC.

Jin *et al. *[18] exploited the time-series of gene expression profiles to determine whether two adjacent interactions are exclusive or cooperative. Each protein has a gene expression time-series such that each interaction is associated with a time-range pair by aligning the corresponding two gene expression time-series of the proteins. Two adjacent interactions are considered to be cooperative and can thus occur simultaneously if their common partner protein has overlapping time-ranges for these two interactions.

### Complex detection using evolutionary information

With the increasing availability of PPI data for most species (such as yeast, fly, worm and so on), it has become feasible to use cross-species analysis to derive insights into the evolution of the PPI networks for complex detection.

Sharan *et al. *recently proposed a series of methods for comparative analysis in two or more species. They used these methods for conserved pathway detection [76], protein function analysis [77] and conserved protein complex detection [78-80]. Basically, to detect the conserved protein complexes in two species, an orthology graph (also called network alignment graph) is constructed, in which each node represents a pair of sequence-similar proteins (homologous or orthology proteins) and each edge represents a conserved interaction between the corresponding protein pairs in each species.

In [78], each node (*u, v *) in the orthology graph is weighted by the sequence similarity between the protein pair *u *and *v *. An edge ((*u*_1_, *v*_1_), (*u*_2_, *v*_2_)) is associated with a pair of weights (*w *(*u*_1_, *u*_2_), *w *(*v*_1_, *v*_2_)), where *w *(*u*_1_, *u*_2_) is the weight of the interaction (*u*_1_, *u*_2_). Two models, the protein-complex model and null model, are proposed to learn the weights of interactions and detect protein complexes in each species. In [79], the researchers detect conserved protein complexes across yeast and fly based on a probabilistic model which considers the evolution of PPI networks through link dynamics and gene duplications [81]. A subgraph in the orthology graph can have a likelihood ratio score from the protein-complex model and null model as above. Candidate subgraphs with highest scores are detected as conserved complexes, using a similar heuristic as in [78].

In [80], a tool called *QNet *was developed for queries in PPI networks. The similarity between two graphs is defined based on the node and edge similarity and the penalty scores for node deletion and insertion. *QNet *then performs tree queries and bounded-treewidth graph queries by the color coding algorithm [82]. Conserved protein complexes are obtained through querying known yeast complexes against the PPI networks of other species.

Another group of researchers, Dutkowski *et al. *[83] also proposed an evolution-based framework to detect conserved protein complexes across multiple species. First, all the proteins from different species are clustered by MCL with BLAST E-scores as pairwise similarity and the proteins from each cluster are homologous and thus believed to have a common ancestral protein. Then, the interactions between the ancestral proteins are assigned weights under the duplication and speciation model of the PPI network evolution. Finally, in the conserved ancestral PPI network, the connected components after removing the edges with weights lower than an appropriate threshold are predicted as conserved protein complexes.

### Complex detection using protein core attachments

In the genome-wide screen for protein complexes using affnity purification and mass spectrometry [2] reported by Gavin *et al. *[3], they observed that the majority of complexes are purified several times and exploited this redundancy to computationally refine the protein complexes. Given a protein pair, a socio-affinity index between them (using a combination of the `spoke' and the `matrix' models) is derived to measure their propensity to be partners. A weighted PPI network is thus constructed, where the edge weight is the socio-affnity index. An iterative clustering is then applied to generate the clusters. Protein complexes generated in this work also support the inherent organization, i.e., core-attachment structures demonstrated in [84].

Zhang *et al. *[67] proposed a dice coeffcient to measure the reliability of protein interactions based on purification records. An unweighted PPI network is constructed by removing unreliable edges with weights less than a pre-defined threshold. Finally, maximal cliques are detected by the maximal clique finding algorithm in [85] and some highly overlapping cliques are merged to form larger dense subgraphs. Predicted complexes in this work are with core-attachment structures. In particular, the core proteins in a detected complex are defined as those present in at least 2/3 of the original cliques which are merged into this complex and the rest are all attachment proteins.

Recently, Leung *et al. *[86] proposed the CORE algorithm, a statistical framework to identify protein-complex cores. The probability for two proteins to be in the same protein-complex core (called the p-value) is mainly determined by two factors: whether these two proteins interact or not and the number of the common neighbors between them. The CORE then calculates the p-values for all pairs of proteins (i.e., pair-wise fashion) to detect cores. The protein-complex cores detected by CORE are non-overlapping. CORE can assign each predicted complex a score to show its probability to be a real complex and then rank all the predicted complexes based on the scores.

**Figure 3:**
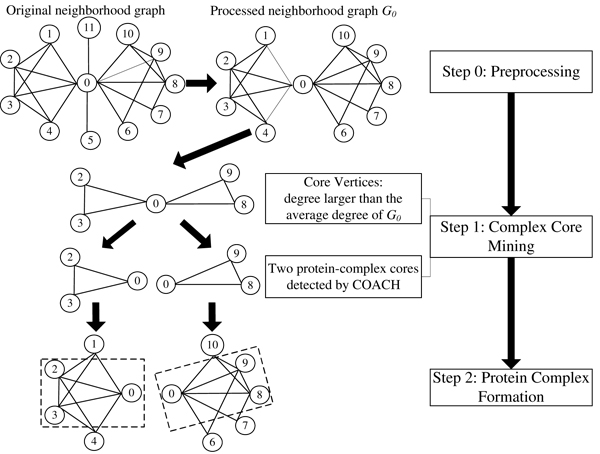
COACH method detects core-attachment complexes with overlapping core structures.

To provide insights into the organization of protein complexes, Wu *et al. *[87] presents a COre-AttaCHment based method (COACH) which detects protein complexes in two stages. In the first stage, COACH defines core vertices from the neighborhood graphs and then detects protein-complex cores as the hearts of protein complexes. In the second stage, COACH includes attachments into these cores to form biologically meaningful structures. Figure 3 illustrates the diagram to detect protein complexes with core-attachment structures. Unlike CORE, the COACH method is able to detect the overlapping cores as shown in Figure 3. In this example, the node 0's neighborhood graph is first constructed and preprocessed. Next, two overlapping complex-cores {0, 2, 3} and {0, 8, 9} are detected. Finally, two complexes, {0, 1, 2, 3, 4} and {0, 6, 7, 8, 9, 10}, are formed by adding attachments into each complex-core.

The ability to detect overlapping cores is essential to understand how different cores are organized into the higher-level structures in PPI networks and how these cores communicate with each other to perform cellular functions. It also facilitates better detection of protein complexes from PPI networks, which will be shown in the evaluation results in the next section.

## Evaluation

Before we present the results of our comparative experiments, let us first introduce the various evaluation metrics that have been used to evaluate their computational methods for complex detection. We will then present the experimental results of comparing different state-of-the-art techniques using these evaluation metrics.

### Evaluation metrics

Overall, there are three types of evaluation metrics used to evaluate the quality of the predicted complexes and compute the overall precision of the prediction methods.

#### Precision, recall and f-measure

Precision, recall and F-measure are commonly-used evaluation metrics in information retrieval and machine learning. For evaluating protein complex prediction, we need to define how well a predicted complex which consists of a set of protein members, matches an actual complex, which is another set of protein members. The neighborhood affnity score *NA *(*p, b *) between a predicted complex *p *= (*V_p_, E_p_* ) and a real complex *b *= (*V_b_, E_b_* ) in the benchmark complex set, as defined in equation (4) below, can be used to determine whether they match with each other. If *NA *(*p, b *) ≥ *ω *, they are considered to be matching (*ω *is usually set as 0.20 or 0.25). Let *P *and *B *denote the sets of complexes predicted by a computational method and real ones in the benchmark, respectively. Let *N_cp_* be the number of predicted complexes which match at least one real complex and *N_cb_* be the number of real complexes that match at least one predicted complex. Precision and Recall are then defined as follows: [39,66,87] (Note that the definition of Recall here is different from the one in [16,27,37]):(4)

,

,

 and 

F-measure, or the harmonic mean of Precision and Recall, can then be used to evaluate the overall performance:

(6) *F *= 2 × *Precision *× *Recall */ (*Precision *+ *Recall *)

#### Sensitivity, positive predictive value and accuracy

Recently, sensitivity (*Sn *), positive predictive value (*PPV *) and accuracy (*Acc *) have also been proposed to evaluate the accuracy of the prediction methods [32,36]. Given *n *benchmark complexes and *m *predicted complexes, let *T_ij_* denote the number of proteins in common between *i ^th^* benchmark complex and *j ^th^* predicted complex. *Sn *and *PPV *are then defined as follows:

(7) and 

Here *N_i_* is the number of proteins in the *i ^th^* benchmark complex.

Generally, high *Sn *values indicate that the prediction has a good coverage of the proteins in the real complexes, while high *PPV *values indicate that the predicted complexes are likely to be true positives. As a summary metric, the accuracy of a prediction, *Acc*, can then be defined as the geometric average of sensitivity and positive predictive value,(8)

#### P-values (functional homogeneity)

As we gained more and more biological knowledge about the proteins, we can associate a protein with (possibly multiple) functional annotations. The statistical significance of the occurrence of a protein cluster (predicted protein complex) with respect to a given functional annotation can be computed by the following hypergeometric distribution in equation (9) [88,89]:(9)

where a predicted complex *C *contains *k *proteins in the functional group *F *and the whole PPI network contains |*V *| proteins. The functional homogeneity of a predicted complex is the smallest p-value over all the possible functional groups. A predicted complex with a low functional homogeneity indicates it is enriched by proteins from the same function group and it is thus likely to be true protein complex. By setting a common threshold which specifies the acceptable level of statistical significance, the numbers of predicted complexes with functional homogeneity under this threshold for the various methods can then be used for evaluating their respective overall performance.

It is important to realize that the evaluation metrics described above can only provide us some sense of how well the current graph mining techniques can be used to detect the protein complexes from protein interaction data. These metrics are by no means  absolute measures — they all have their own limitations, especially for sensitivity (*Sn *), positive predictive value (*PPV *) and Accuracy (*Acc *). For sensitivity (*Sn *), if a method predicts a giant complex which covers many proteins in the known real complex set, then this method will get a very high *Sn *score. As for PPV value (*PPV *), it does not evaluate overlapping clusters properly. Here is a case in point: if the known gold standard MIPS complex set (with proteins that belong to multiple complexes) [90] is taken to match with itself, then the resulting *PPV *value is 0.772 instead of 1 (indicating an imperfect match) while the Precision and Recall are both correctly 1. As such, the Accuracy (*Acc *) score, as the geometric average of *Sn *and *PPV *, will also not make good sense. In addition, all the evaluation metrics described above assumed that a complete set of true protein complexes is available, where in reality we are far from it. If a method predicts an unknown but real protein complex (which is not similar with any of the known complexes), all of these evaluation metrics will regard it as a false positive. Furthermore, for P-values, since its calculation relies on the availability of the proteins' functional information, its applicability would be limited in the less studied genomes. As such, so far it has mainly been used in the model organism yeast for which rich molecular functional information is available.

Relatively speaking, the Precision, Recall, F-measure and P-values are thus more acceptable for evaluating the performance of current techniques. Still, we need to treat the current evaluation metrics with caution, as more research is needed to come up with a robust evaluation metric for the protein complex prediction task.

### Comparative evaluations

For this review, we have performed extensive experiments to compare the existing techniques for which we are able to obtain the software implementations — either source codes or binary executable systems. Those existing techniques that do not provide available software are not included in the comparison exercise. Fortunately, we have a good representative collection of implemented algorithms for comparison: MCODE [27], RNSC [57], MCL [31], DPClus [38], CFinder [43], DECAFF [16], CORE [86] and COACH [87]. Note that for fair comparisons, we have turned off the filtering step for the DECAFF and RNSC methods because they made use of the functional information to filter away possible false positive complexes while the other techniques only used topological information. For the experiments, we have used the default values for their parameters of all these methods as provided by the software (CORE and CFinder have no parameters). Clearly, some methods could have achieved better results by further tuning of their parameters — however, there is no principled way to set the reasonable values for these parameters other than using their default values.

In order to evaluate the predicted complexes, the set of real complexes from [36] was selected as the benchmark. This benchmark set consists of 428 gold standard protein complexes, which are from three main sources: (I) MIPS [90], (II) Aloy et al. [91] and (III) SGD database [92] based on Gene Ontology (GO) annotations. In our experiments, *ω *is set as 0.20 to evaluate if a predicted complex matches with a gold standard protein complex (see equations (4) and (5)).

We have compared these techniques over two publicly available benchmark yeast PPI datasets, namely DIP data [93] and Krogan data [33]. DIP (the Database of Interacting Proteins) consists of 17203 interactions among 4930 proteins. Krogan PPI data consists of 14077 high-quality interactions involving 3581 proteins (with a cut-off of 0.101 as shown in their supplementary Table S8 [33]).

Tables 1 and 2 show the detailed comparative results of the various computational detection methods on the DIP data and the Krogan data, respectively. For each detection method, we have listed the number of complexes predicted (# complexes), the number of proteins covered by the predicted complexes (# covered proteins), the number of predicted complexes which match at least one real complex (*N_cp_*) and the number of real complexes that match at least one predicted complex *N_cb_*. Taking MCODE on DIP data as an example, it has predicted 50 complexes, of which 44 match 21 real complexes. These 50 predicted complexes cover 844 proteins out of 4930 proteins in DIP. As shown in these two tables, MCL and RNSC assigned every protein (4930) into its predicted complexes as long as they are present in PPI networks (they also predicted much more complexes than the other methods) while all the other methods only assigned those highly interactive proteins (or the proteins that occurred in the dense subgraphs) into the predicted complexes. In fact, both MCL and RNSC basically partitioned the PPI network simultaneously into non-overlapping clusters while the remaining approaches are more sensible by generating clusters in a one-by-one manner and allowing overlaps in the clusters/complexes. We also noticed that for DECAFF algorithm the number of predicted complexes that matches at least one real complex (*N_cp_*) is significantly higher than the other methods — this is mainly because it is designed to search many dense and possibly overlapping complexes from the PPI networks.

**Table 1:** Results of various approaches using DIP data

Algorithms	MCODE	MCL	RNSC	COACH	CORE	DECAFF	CFinder	DPClus
# complexes	50	1246	2435	746	1722	2190	245	1143
# covered proteins	844	4930	4930	1838	3777	1832	2008	2987
*N_cp_*	21	212	234	285	221	605	84	193
*N_cb_*	44	256	289	249	256	243	111	274

**Table 2:** Results of various approaches using Krogan et al.'s data

Algorithms	MCODE	MCL	RNSC	COACH	CORE	DECAFF	CFinder	DPClus
# complexes	52	834	1890	570	1232	2143	122	689
# covered proteins	651	3581	3581	1428	2665	1478	1578	1996
*N_cp_*	29	147	245	244	201	759	45	167
*N_cb_*	45	197	283	193	229	192	63	241

**Figure 4:**
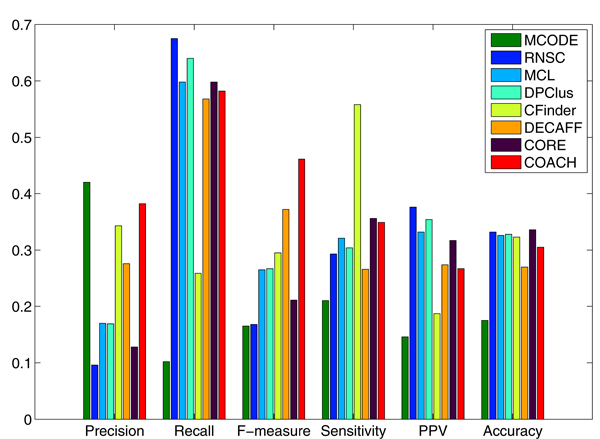
**Comparative performance of existing methods in terms of various evaluation metrics for DIP data.** The methods are ordered chronologically in the years in which they were published.

**Figure 5:**
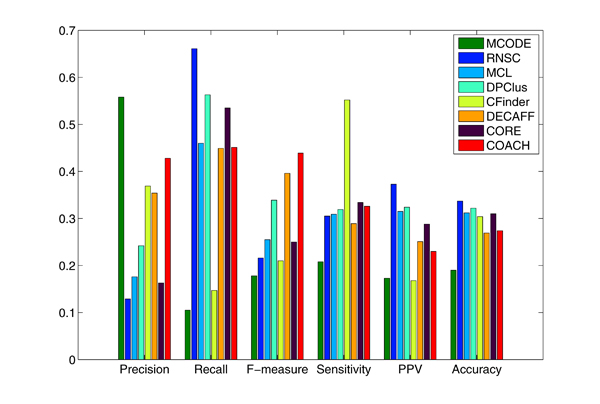
**Comparative performance of existing methods in terms of various evaluation metrics for the Krogan data.** The methods are ordered chronologically in the years in which they were published.

Figures 4 and 5 show the overall comparison results of existing methods in terms of various evaluation metrics, including Precision, Recall, F-measure, Sensitivity, PPV and Accuracy for DIP and Krogan data, respectively — we have also included the Sensitivity, PPV and Accuracy measures here for completeness. In general, we can focus on the F-measures. It is heartening to note that the prediction approaches have improved in performance over the years (the methods were ordered chronologically in the years in which they were published). In Figure 4 , we observe that MCODE was able to achieve the highest precision in DIP data. However, similar to CFinder, it actually predicted very few protein complexes (only 50 for MCODE and 245 for CFinder) and also matched with fewer real complexes than the other methods (as shown in Table 1), resulting in their much low recall and F-measure values. We noticed that CFinder attained an unusally higher sensitivity than other methods. This is actually because it predicted an impossibly huge cluster which contains 1417 proteins; as such, all the proteins in the benchmark complexes were pretty much covered by this very big cluster, giving a very high sensitivity value for CFinder. We do not think we should give CFinder a high rating in performance because of this. MCL, RNSC, CORE, DECAFF, DPClus are observed to have attained high recall values by correctly matching many real complexes. Unfortunately, because their precision is low, they end up with relatively lower F-measures. Overall, COACH achieved the highest F-measure due to its balanced precision and recall. The results for Krogan data in Figure 5 are similar to those in Figure 4 (DIP data), suggesting consistency in the evaluation results.

**Figure 6:**
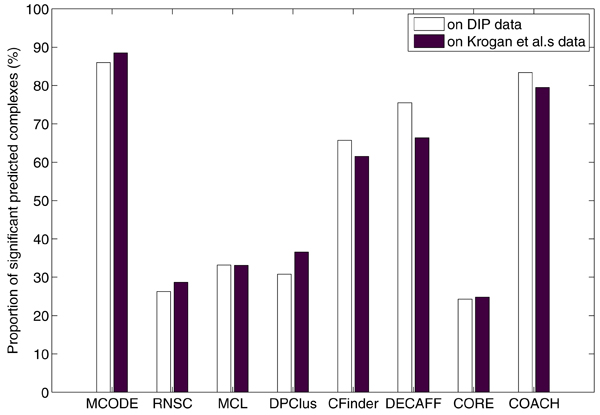
Proportion of statistically significant complexes predicted by various methods in terms of P-values.

Figure 6 shows the relative performance of the methods in terms of P-values. We calculated the P-values with Bonferroni correction for predicted complexes using the tool SGD's GO::TermFinder (http://db.yeastgenome.org/cgi-bin/GO/goTermFinder.pl). The complexes with only one protein are discarded because calculating P-values for those complexes makes no sense according to the equation (9). We considered a predicted complex with a corrected P-value ≤ 0.01 to be statistically significant. The results showed that MCODE was able to obtain the highest proportion of significant complexes.

Unfortunately, this was an artefact of its predicting very few complexes as compared to all the other methods. Ignoring MCODE, then COACH and DECAFF have both achieved decent proportions of their predicted complexes as significant. As for DPClus, MCL and RNSC, because they predicted many protein complexes with extremely small size (e.g., with only two proteins) which resulted in large P-values since they could occur by chance. For CORE, it generated many protein-complex cores with only one protein. Given such a core with one protein, CORE can only form a protein complex by including all the interacting partners of the protein as attachment. These protein complexes have low statistical significance, leading to the low performance of CORE.

## Discussions and conclusions

Identifying protein complexes is important for biological knowledge discovery since many important biological processes in the cell are carried out through the formation of protein complexes. However, there is currently a wide gap between data on protein complexes and (pairwise) protein-protein interactions. High throughput technologies for detecting pairwise protein-protein interactions *en masse *have already become routine in the laboratories for generating large datasets of protein interaction data , while the high-throughput technologies for detecting protein complexes remained relatively immature. Hence computational approaches for detecting protein complexes are needed to help fill up the relatively empty map for the protein "complexome".

In this paper, we have reviewed current computational approaches that have been proposed to exploit the abundant protein interaction data to bridge the data gap for protein complexes. Protein interaction graph mining algorithms that identify graphical subcomponents in the protein-protein interaction networks can be used for predicting protein complexes. We have surveyed the state-of-the-art algorithms by describing the traditional graph clustering methods as well as the recent emerging techniques for computational detection of protein complexes from PPI and other data sources. Table 3 lists five methods that, in our opinion, represent the key developments for computational protein complex detection so far. Our experimental results indicate that predicted complexes were shown to match or overlap reasonably well with the known protein complexes in our benchmark database. This suggests that the computational methods can help biologists in their continuing search for new protein complexes.

**Table 3:** Key milestones of computational protein complex detection

Methods	Main contributions
MCODE [27]	MCODE pioneered the computational detection of protein complexes from PPI networks.
MCL [30,31]	MCL is a widely used method [33-36] with good convergence and robustness [32]
Krogan et al. [33]	They provided and exploited a comprehensive TAP dataset for computational protein complex detection.
Gavin et al. [3]	In addition to providing a TAP dataset widely used for protein complex detection, they described the inherent organization of protein complexes with core-attachment structures.
Sharan et al. [78-80]	They systematically conducted cross-species analysis of PPI networks to derive evolutionary information for protein complex detection.

On the other hand, our results also show that more further research is needed to improve these methods. In the following discussions, we describe three key challenges for further improvements [94].

### PPI data is noisy with high false positive and false negative rates [95-97]

The computational methods are highly dependent on the quality of the underlying interaction data. Unfortunately, despite the abundance of PPI data, the data quality of these data still leaves much to be desired. For example, the experimental conditions in which the PPI detection methods are carried out may cause a bias towards detecting interactions that do not occur under physiological conditions, resulting in false positive detection rates that could be alarmingly high [98]. At the same time, the high-throughput methods can also fail to detect various types of interactions, e.g. loss of weak transient interactions, loss of post-translational modification and bias against soluble or membrane proteins [10]. These results in high false negative rates and low experimental coverage of the interactomes. The computational methods for complex mining are clearly limited by the poor quality of the underlying PPI data. Further improvements for complex mining can be obtained by improving the quality of the PPI data with new and more powerful experimental detection technologies, or with computational approaches for validating the existing protein interactions (to address false positive interaction issue) and predicting novel protein interactions (to address false negative interaction issue).

### Graph mining of PPI networks is intrinsically computationally challenging

PPI networks are very large graphs with thousands of vertices and tens of thousands of edges, even for a simple organism such as yeast. For the more complicated species such as the human being, the scale and complexity of the PPI networks are clearly overwhelming. Graph mining on the PPI networks is certain to test the limits of any computational methods. The fact that many graph-based problems, such as subgraph isomorphisms [99,100] and enumeration [101,102], are NP-hard [103,104], indicates that mining the PPI networks is intrinsically computationally challenging. Although mining objects such as complexes from protein interaction networks are computationally challenging problems, it may be possible to reduce the search space and time complexity and obtain better mining results by exploiting biological knowledge coupled with the development of novel graph mining techniques that are specialized on such networks [105].

### Integrate various biological evidences into the mining process

Through integrating various independently obtained biological evidences, we can assess/weight the protein interactions by using appropriate confidence measures. For example, we can employ metrics from biological evidences such as reproducibility of the interactions from multiple experimental methods, support from such other non-interaction data as co-expression, co-localization and shared functions, as well as the conservation of the protein interactions across other genomes, etc to address the limitations in the current quality of PPI data. Computational methods, such as Bayesian network models [50] and kernel methods [106], have been proposed to integrate different biological resources (e.g. integrate weighted genomic features in [50] and mapping different features into high dimensional vector space in [106]).

From this review, it is clear that researchers have been tireless in devising new computational approaches for detecting protein complexes. It is indeed heartening that our evaluation results have showed that the proposed methods have generally improved in performance over the years. In time, we will be able to fill up the currently rather empty map of the complexome with combined efforts  from biologists as well as computational scientists computational scientists.

## Competing interests

The authors declare that they do not have any competing interests.

## Authors contributions

XL and MW drafted the manuscript together. MW was responsible for performing experiments to compare the existing techniques. CKK and SKN participated in discussion and conceptualization as well as revising the draft. All authors have read and approved the manuscript.
